# Label-free fluorescence lifetime imaging for the assessment of cell viability in living tumor fragments

**DOI:** 10.1117/1.JBO.29.S2.S22709

**Published:** 2024-06-14

**Authors:** Jason T. Smith, Chao J. Liu, Jeannine Degnan, Jonathan N. Ouellette, Matthew W. Conklin, Anna V. Kellner, Christina M. Scribano, Laura Hrycyniak, Jonathan D. Oliner, Chris Zahm, Eric Wait, Kevin W. Eliceiri, John Rafter

**Affiliations:** a Elephas, Madison, Wisconsin, United States; b Booz Allen Hamilton, McLean, Virginia, United States; c Center for Quantitative Cell Imaging, Madison, Wisconsin, United States

**Keywords:** fluorescence lifetime, fluorescence lifetime microscopy, multiphoton, metabolism, necroptosis, apoptosis, cell death, nicotinamide adenine dinucleotide phosphate, cancer

## Abstract

**Significance:**

To enable non-destructive longitudinal assessment of drug agents in intact tumor tissue without the use of disruptive probes, we have designed a label-free method to quantify the health of individual tumor cells in excised tumor tissue using multiphoton fluorescence lifetime imaging microscopy (MP-FLIM).

**Aim:**

Using murine tumor fragments which preserve the native tumor microenvironment, we seek to demonstrate signals generated by the intrinsically fluorescent metabolic co-factors nicotinamide adenine dinucleotide phosphate [NAD(P)H] and flavin adenine dinucleotide (FAD) correlate with irreversible cascades leading to cell death.

**Approach:**

We use MP-FLIM of NAD(P)H and FAD on tissues and confirm viability using standard apoptosis and live/dead (Caspase 3/7 and propidium iodide, respectively) assays.

**Results:**

Through a statistical approach, reproducible shifts in FLIM data, determined through phasor analysis, are shown to correlate with loss of cell viability. With this, we demonstrate that cell death achieved through either apoptosis/necrosis or necroptosis can be discriminated. In addition, specific responses to common chemotherapeutic treatment inducing cell death were detected.

**Conclusions:**

These data demonstrate that MP-FLIM can detect and quantify cell viability without the use of potentially toxic dyes, thus enabling longitudinal multi-day studies assessing the effects of therapeutic agents on tumor fragments.

## 1 Introduction

Drug development is currently arduous and costly.^1^^,^^2^ Therefore, rapid, cost-effective evaluation of drug candidates in translationally relevant *in vitro* model systems is highly desirable. Concurrently, tumor and patient heterogeneity present a difficult barrier to accurate drug efficacy determination. For this, patient-specific assays are currently under development. *In vitro* models with increasing translational relevance include spheroids, organoids, and excised primary tumor tissue. Of these models, primary tumor tissue is recognized as the most desirable for preserving both cellular heterogeneity and structural elements of the tumor microenvironment (TME). To evaluate drugs in these representative models, microscopy techniques can provide analytical information with spatial context on a cellular level.

Advances in optical microscopy allow for assessing biomolecular characteristics of living cells in a label-free manner within intact tissues without disruption of the three-dimensional (3D) TME. To this end, various imaging technologies have been employed to monitor the morphological and chemical changes in response to external stress agents. Two more recent developments in the field include the use of coherent anti-Stokes Raman scattering, which has been used to profile treatment response in live tissue based on changes in endogenous vibrational contrast (e.g., increased number of cytoplasmic lipid droplets due to drug-induced stress),^3^^,^^4^ and dynamic optical coherence microscopy, which has been used to measure changes in intracellular motion brought about by loss of metabolic activity as a determination of cellular viability.^5^^,^^6^ In addition, autofluorescence imaging of endogenously fluorescent metabolic co-factors nicotinamide adenine dinucleotide phosphate [NAD(P)H] and flavin adenine dinucleotide (FAD) has been used for decades to monitor glycolysis and identify potential cancer tissue.^7^^–^^10^ The observation of cancers’ tendency to favor glycolytic activity in the presence of oxygen was made by Otto Warburg with a practical fluorescence application realized by employing the Chance ratio—[FAD]/([FAD]+[NAD(P)H])—to monitor glycolytic activity in cells.^11^^–^^13^ With the advent of multi-channel multiphoton fluorescence lifetime imaging microscopy (MP-FLIM), this method has found increasing use in providing sensitive information regarding the metabolic status of living cells in tissue in a highly direct fashion.^14^^–^^16^ Specifically, MP-FLIM can monitor disruption of the cell associated with either induced or stochastic programmed cell death by monitoring lifetime changes in metabolic co-factors.^17^^–^^20^

Probing the cellular metabolism of primary living tumor fragments in response to therapy via label-free molecular imaging allows for early response monitoring without the negative effects of exogenous labels.^10^ However, only imaging approaches capable of reaching depths of 50  μm or greater within living tumor fragments are desirable to ensure tissue with intact cellular architecture is probed.^21^ In addition, it is highly desirable to monitor longitudinal changes in metabolism in response to drug treatment using less disruptive microscopy techniques. For both these reasons, MP-FLIM is uniquely suited to provide spatial metabolic insight from intrinsic molecules with cell resolution deep within living tissue in a non-destructive manner. Unlike traditional fluorescence or confocal microscopy, multiphoton microscopy (MPM) utilizes infrared (IR) light to excite fluorescent molecules. For NAD(P)H and FAD, the use of IR light is particularly advantageous as it can penetrate up to two to four times deeper into tissue samples relative to the shorter, higher energy photons needed in traditional fluorescent techniques to generate data from these molecules. Furthermore, when detecting fluorescent signals, MPM is not hampered by confocal optical elements that screen out scattered emitted light and can maintain subcellular spatial resolution to deliver quantitative data deeper into living tissue than other techniques.^22^^,^^23^ For living tissue applications, it is the preferred technique because it has been shown to be less deleterious to tissue than techniques which employ higher energy light sources, particularly in tissue adjacent to the focal plane.^24^^,^^25^ This makes it ideal for applications requiring repeated tissue imaging to monitor potential drug activity over time. By imaging intrinsic metabolic co-factors, assays employing intrinsic signals can be performed without introducing potentially toxic extrinsic labels, thereby facilitating longitudinal studies.

While the MP-FLIM techniques described here can be applied to many different *in vitro* models, it is becoming increasingly clear that it is most desirable to study primary human cancer tissue which preserves the native cellular makeup and morphology of the TME. Spheroid and organoid models of human cancer have notable drawbacks. For example, not all cancer cell lines can be cultured to make spheroids.^26^ In addition, even when spheroids can be derived from patient cancer cells, they intrinsically lack essential features of the TME without implementing complex multi-region biopsy preparation protocols^27^ and have limited potential to investigate anti-tumor immune response.^28^^,^^29^ The MP-FLIM assays described here were optimized for freshly harvested tumors. These tumors are cut into live tumor fragments (LTFs) which are subsequently assayed individually *ex vivo*. Unlike spheroids or organoids, which do not preserve the native TME, there is no requirement for the expansion of cells or reconstitution of immune cells—enabling a faster turnaround time to characterize the efficacy of candidate therapeutics.

Herein, for the first time, we present a spatial assay to monitor baseline viability and therapeutic efficacy in LTFs based on MP-FLIM and fully validate it against exogenous ground-truth labels. The approach is characterized using LTFs derived from two syngeneic mouse cancer models and across a range of cell death pathways—ranging from apoptosis to necroptosis. In addition, we demonstrate our method’s clinical potential by assessing chemotherapeutic efficacy over the duration of 48 h. Importantly, the deep optical sectioning and the label-free nature of this MP-FLIM metabolic assay leave the LTFs intact and viable for downstream assays (e.g., cytokine profiling, immunohistochemistry, messenger ribonucleic acid expression). Altogether, this work highlights the unique potential of these imaging capabilities to accelerate drug discovery and facilitate better prognostic outcomes.

## 2 Materials and Methods

### 2.1 Syngeneic Tumor Implantation, Harvesting, and Processing

All animal procedures were conducted with the approval of the Institutional Animal Care and Use Committee at Elephas Bioscience. Animal facilities have been accredited by the American Association for Accreditation for Laboratory Animals Care International. Tumor xenografts were generated by subcutaneous flank injection of $1\times 10^{6}$ CT26 or $0.5\times 10^{6}$ MCA205 cells in phosphate-buffered saline (PBS) into 8 to 16-week-old athymic nude mice (BALB/c or C57BL/6 for CT26 or MCA205, respectively). Tumors were monitored daily for more than 3 to 4 weeks and harvested at a target size of ∼200 to $350\;{\mathrm{mm}}^{3}$. Tumor excisions were processed within 60 min of harvesting using a proprietary 3D cutting instrument (Elephas Bioscience, Madison, Wisconsin, United States). The tissue was cut into small fragments measuring $300\times 300\times 300\;{\mu m}^{3}$ or $900\times 900\times 300\;{\mu m}^{3}$.

### 2.2 Cell Culture and Reagents

For culture and subsequent imaging, CT26 and MCA205 fragments were placed into eight well IBIDI chambered coverslip slides (IBIDI, #80807, IBIDI, Fitchburg, Wisconsin, United States) containing 200  μL mouse complete microscopy medium—phenol red-free Roswell Park Memorial Institute Medium (RPMI) 1640 (ThermoFisher, Waltham, Massachusetts, United States, #11835030)—supplemented with 10% FBS (Fisher Scientific, # 10082147, Hampton, New Hampshire, United States), 10 mM HEPES (Gibco, Hampton, New Hampshire, United States, 15630080), 1 mM sodium pyruvate (Sigma-Aldrich, # S836, St. Louis, Missouri, United States), 1X MEM non-essential amino acids (Sigma-Aldrich, # M7145), 1X GlutaMAX (Thermo, # 35050061), and 10,000  U/mL penicillin-streptomycin (Gibco, # 15140122).

### 2.3 On-Stage Sample Incubation

An electric top-stage incubation system (H301-T-UNIT-BL-PLUS, Okolab, Pozzuoli, Italy) was used to maintain a 37°C sample environment inside the sample chamber (H301-K-FRAME, Okolab). In addition, a humidity controller (HM-ACTIVE, Okolab) was used to maintain 95% humidity. Furthermore, a gas controller (${\mathrm{CO}}_{2}\text{-}O_{2}$-UNIT-BL [0-20; 1-95], Okolab) was used to maintain 5% ${\mathrm{CO}}_{2}$. Background air from an air pump (OKO-AIR-PUMP-BL, Okolab) was used to maintain 21% $O_{2}$. The eight well IBIDI chambered coverslip slides containing fragments were placed in the stage incubator for 1 h prior to imaging the sample.

### 2.4 Fluorescent Probes, Chemical Reagents, and Sample Treatment Protocols

#### 2.4.1 Fluorescent probes

Propidium iodide (PI) was used at a final working concentration of 25  μM in RPMI complete (phenol red-free) media. Caspase 3/7 Red (IncuCyte) was diluted in fragment culture media to a final concentration of 0.75×.

#### 2.4.2 Chemical reagents

Hydrogen peroxide ($H_{2}O_{2}$) (#968804, Walgreens, Deerfield, Illinois, United States) was used at 200  μM working concentration. A 3 mg of shikonin powder (#6829, Tocris, Bristol, United Kingdom) was added to 1 mL dimethyl sulfoxide (DMSO) and vortexed until dissolved. A 10  μL of shikonin + DMSO mixture was added to 90  μL of 1× PBS (stock concentration 0.1  mg/mL). Fifty microliters of this was added to 50  μL RPMI complete (50  μg/mL diluted stock concentration). Stock solution was diluted to yield a final 7.5  μg/mL working concentration.^30^ A 10  μL of 10 mM staurosporine (HY-15141, MedChem Express, Princeton, New Jersey, United States) was diluted into 100  μL of sterile DMSO and 890  μL of 1× PBS (final concentration 0.1 mM). The stock solution was diluted to yield a final 2  μM working concentration. A 2.5 mg of 3-myethyladenine (3-MA) (M9281, Sigma-Aldrich) was dissolved in 2.5 mL (DMSO). Ten microliters of this was diluted into 90  μL of RPMI complete (670  μM diluted stock concentration). This was diluted to yield a final working concentration of ≈25  μM. Five milligrams of doxorubicin (D1515, Sigma-Aldrich) was dissolved in 1 mL DMSO and 5 mL sterile RPMI. This stock was diluted to yield a final 30  μM working concentration.

#### 2.4.3 Heat shock protocol

Six murine LTFs were pipetted into a 1.5 mL Eppendorf tube (33.3  μL per fragment, 200  μL RPMI total). These tubes were placed on a heat block that was pre-heated to 65°C for 5 min. Then, the vial was put onto ice for 2 min. Afterward, the vial was placed at 37°C for 5 min.

### 2.6 Multiphoton Lifetime Imaging Instrumentation

LTFs were imaged on a custom-built inverted multiphoton photon fluorescence microscope (Bruker, Billerica, Massachusetts, United States) using a 20× air objective (NA = 0.8, Zeiss, Oberkochen, Germany). A titanium:sapphire pulsed (80 MHz) laser (Chameleon, Coherent Inc., Santa Clara, California, United States) was used for excitation, and three GaAsP H10770B-40 photomultiplier tubes (Hamamatsu, Shizuoka, Japan) were used for spectral fluorescence collection across three separate wavelength bands: (i) 460±25  nm (Chroma, Irvine, California, United States, HQ460/50m), (ii) 525±25  nm (Chroma HQ525/50m), and (iii) 618±25  nm (Semrock, Rochester, New York, United States FF01-618/50). An excitation wavelength of 740 nm was used to measure NAD(P)H autofluorescence as well as PI and Caspase 3/7 Red signal. In addition, an excitation wavelength of 880 nm was used to image FAD autofluorescence. Time-correlated single-photon counting electronics (Time Tagger Ultra, Stuttgart, Germany) were used to acquire fluorescence lifetime images for more than a 30 s acquisition timeframe (≈60  s total for two excitation wavelengths). A pixel dwell time of 2  μs was used to acquire 512×512  pixel images.

Retrieval of the spectral MP-FLIM apparatus’ instrument response function (IRF, $\tau_{\mathrm{eff}}\approx 0\;\mathrm{ns}$) was obtained by measuring instantaneous scattering from gold nanoshell samples. To prepare the gold nanoshell (GSCR150, nanoComposix, San Diego, California, United States) sample for IRF measurement, 20  μL of the homogenized solution is placed onto a glass bottom 35 mm petri dish (MatTek Life Sciences, Ashland, Massachusetts, United States) and allowed to dry. Afterward, the edge of this sample is focused on by the microscope, allowing an IRF to be captured across all relevant excitation wavelengths and spectral acquisition channels.

### 2.7 FLIM Data Preprocessing

Photon count thresholding using NAD(P)H fluorescence intensity obtained by summing over time was undertaken to remove pixels with exceedingly low signal-to-noise ratio (SNR) from subsequent analysis. Hence, only the pixels with sufficient photon counts (n>20) were used in subsequent analysis, and all other pixels (e.g., empty space beyond the fragment periphery) were discarded. Afterward, spatial binning using either a 5×5 or a 6×6 circular kernel^31^ was used to increase the photon counts of FLIM data of NAD(P)H and FAD, respectively, prior to phasor quantification.

### 2.8 FLIM-Phasor Analysis

Details regarding the phasor quantification workflow used herein are provided in great depth elsewhere.^32^ The first harmonic frequency f [see Eq. (1)] was used for all analysis herein (1/D≈80  MHz)—where D is the period of the recorded decay.

$$z(x,y)=\frac{\sum_{p=1}^{G}G_{p}(x,y)e^{i2\pi ft_{p}}}{\sum_{p=1}^{G}G_{p}(x,y)},$$ (1)

where f is the phasor frequency, G is the number of time bins, and $G_{p}$ is the p’th intensity value at pixel (x,y).

### 2.9 Phasor Calibration

Briefly, the uncalibrated phasor of the IRF data corresponding to the sample of interest (i.e., gold nanoshells—preparation steps detailed previously) was calculated using Eq. (1) by taking the sum over many pixels ($n_{pixels}$ > 500) located along the edge region of the gold nanoshell sample. This calibration phasor, $z_{\mathrm{IT}}$, associated with lifetime $\tau_{\mathrm{IRF}}=0\;\mathrm{ns}$, was retrieved for all three PMTs and for both excitation wavelengths used.

Following IRF calibration, the resulting phasors of single-exponential decays of known samples (e.g., fluorescein isothiocyanate, FAD, coumarin) were located on the universal semicircle (or universal circle) to confirm the accuracy of the instrument.^33^

### 2.10 Phasor Ratio Calculation

In the case of decays comprised of fluorescence contribution originating from two or more different species, such as in the mixture of free and bound NAD(P)H components encountered herein, the resulting phasor of the mixture is a linear combination of the phasor of each species.^33^^–^^35^ The intensity fraction of each species in the mixture can be recovered from the location of the phasor with respect to the pure species (reference) phasors. In the cases studied here, the NAD(P)H and FAD reference lifetimes can be inferred from the observed linear arrangement of the phasors—which were in agreement with reported values.^36^ Because of shot noise and other sources of variance (e.g., differing bound state populations of NAD(P)H),^37^ orthogonal projection of each phasor onto the line connecting both references is undertaken to obtain the phasor ratio r expressing the relative distance of the phasor to the two reference locations, as follows:

$$\left\{ \begin{array}{l} r=\frac{g-g_{2}}{d}\;\cos(\theta )+\frac{s-s_{2}}{d}\;\sin(\theta )\\ d=((g_{1}-g_{2}{)}^{2}+(s_{1}-s_{2}{)}^{2}{)}^{1/2}\\ \sin(\theta )=\frac{s-s_{2}}{d},\cos(\theta )=\frac{g-g_{2}}{d} \end{array},\right.$$ (2)

where $\left(s_{i},g_{i}\right)$, i=1 or 2, corresponds to the phasor of the two references and (g,s) is the multi-component phasor. The phasor ratio r defined in Eq. (2) corresponds to the intensity fraction of the short-lifetime species in the mixture.^38^

### 2.11 Lifetime Metabolic Ratio (LMR) and Normalized Metabolic Shift (NMS) Quantification

For autofluorescence FLIM of NAD(P)H, unbound NAD(P)H has a lower lifetime than its protein-bound counterpart (≈0.4 and 3.5 ns, respectively). However, for FAD, the opposite is true. To take full advantage of valuable information regarding metabolic co-factors, a ratio of the bound fraction of NAD(P)H and FAD has demonstrated powerful utility.^22^^,^^23^^,^^39^^–^^41^ Herein, this metric is referred to as the LMR—the equation for which is simply stated as

$$\mathrm{LMR}=\frac{1-r_{\mathrm{NADH}}}{r_{\mathrm{FAD}}}.$$ (3)

Previous work has demonstrated the use of baseline normalization—that is, measurement of relative shifts in FLIM signature compared with baseline over time—as an indicator of metabolic perturbation in response to treatment.^42^^,^^43^ Herein, normalization of the LMR metric described above was undertaken by subtraction with the mode of the LMR at $t_{0}$ (i.e., prior to undergoing any treatment). Given that metabolic shifts can be a powerful predictive measure of cell status, we have called this normalized shift in the LMR the NMS in the following equation:

$$\mathrm{NMS}=\mathrm{LMR}(t_{n})-\mathrm{mode}\;(\mathrm{LMR}(t_{0})).$$ (4)

**Fig 1:**
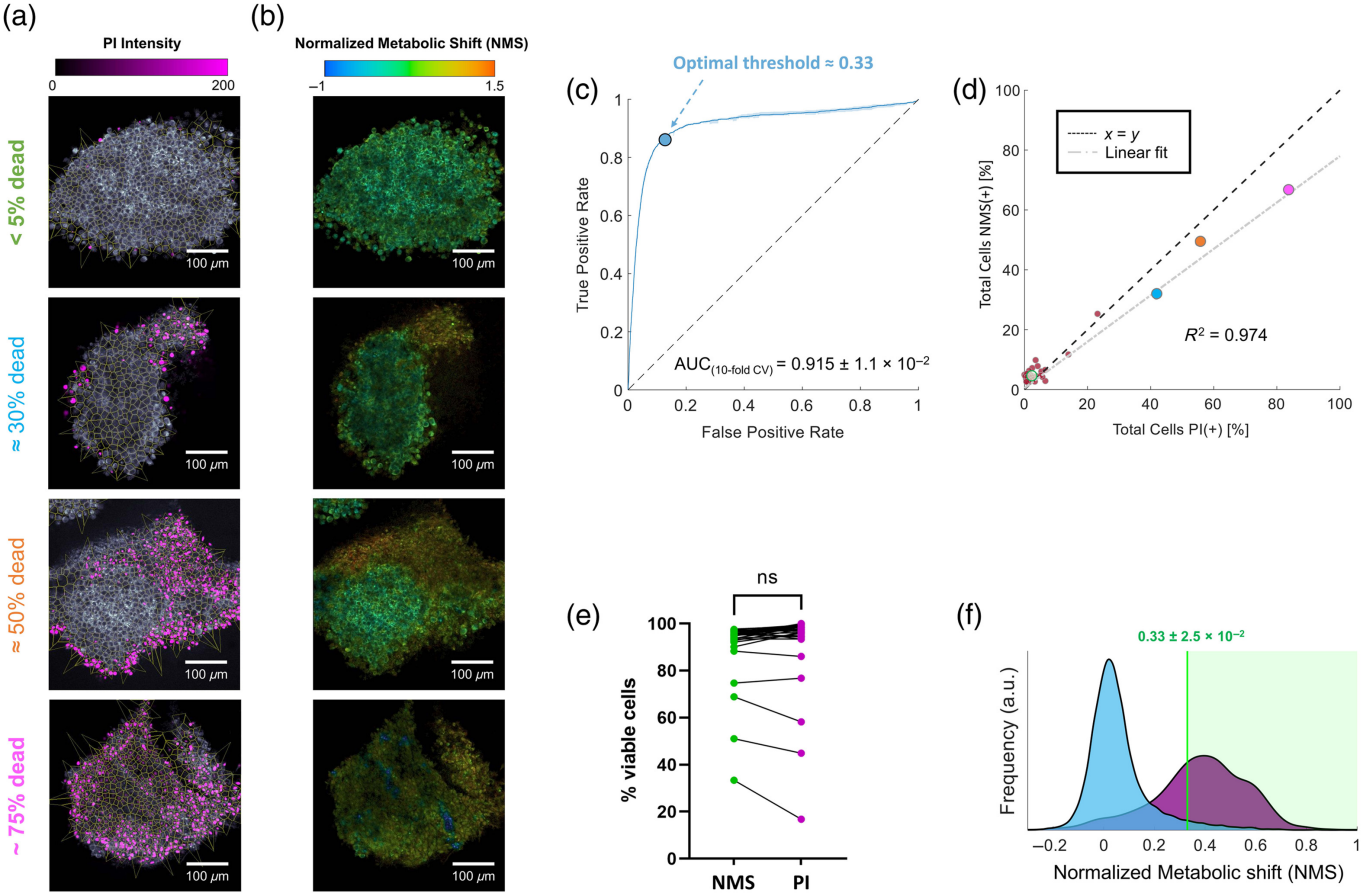
Baseline viability screening of CT26 LTFs. (a) PI intensity images overlaid with Voronoi segmented cells. (b) Corresponding NMS images. (c) Ten-fold cross-validation AUROC analysis using the NMS KDEs from panel (f) (n>16,000 cells). (d) Percentage of cells marked dead using PI (horizontal axis) versus NMS (vertical axis) across 30 fragments. While most fragments contained >90% living cells, we chose four fragments which represent increasing levels of PI staining, represented with the specific color scheme outlined in panel (a) (leftmost text). (e) Side-by-side comparison of viability obtained using the NMS versus PI (n=30). (f) Probability distribution functions of NMS obtained for all PI-positive cells versus PI-negative cells across all 30 fragments (n>16,000 cells). Average and standard deviation optimal threshold values were retrieved for each fold via AUROC analysis and are visualized by a vertical green solid line in panel (f).

Hence, in all cases with exception of the viability screen (Fig. 1), where the mode across all single-cell LMR was used for this normalization, all NMS results detailed herein were obtained using a baseline measurement of the same fragment prior to the addition of any treatment. An example of this is illustrated in Fig. 5 and Figs. S1–S3 in the Supplementary Material. 

### 2.12 Statistical Analysis

PI labeling is highly variable depending on the cell death mechanism observed (e.g., necroptosis dispersing PI over a larger cellular surface area than that measured after apoptosis). Hence, to perform a single-cell analysis of FLIM signature compared with PI labeling, the determination of single-cell regions was necessary. Given the lack of clear cell boundaries using autofluorescence intensity alone within our highly dense LTF samples, manual identification of cell nuclei was undertaken for all single-cell analyses detailed herein. The centroid of each nuclei region was then used for Voronoi segmentation. To ensure Voronoi cells that contained large regions of noncellular space were not included in the analysis, all regions with a total number of pixels larger than 1000 were excluded. The remaining Voronoi cells were used to calculate the average NMS and total summed intensity of PI. Given the slight presence of (likely) non-PI fluorescence background in the red emission channel meant to collect PI, a total intensity value of higher than 1000 photon counts across the entire Voronoi cell was used to mark cell death via PI staining. Given the large number of factors affecting fluorescence intensity readings from experiment to experiment (e.g., PI concentration, differing tissue-dependent diffusion kinetics, variations in optical properties, imaging parameters), this threshold is subject to enormous variability. However, when sufficient photon signal is collected from samples of interest, these factors do not affect autofluorescence lifetime measurements to the same degree. Caspase 3/7 Red was used to extrinsically confirm our caspase-dependent [e.g., staurosporine or heat-shock (apoptosis)] or independent [e.g., shikonin (necroptosis) or $H_{2}O_{2}$ (necrosis)] cell death mechanisms.

Kernel density estimation (KDE) distributions were calculated in MATLAB using the lower and upper bounds of the listed x-axis and a $5\times 10^{-3}$ interval [e.g., −0.5:0.005:1 for Fig. 1(d)] for all cases herein. Each probability distribution function herein was normalized such that the sum over all the distribution equals one.

## 3 Results

### 3.1 Label-Free Screening of Baseline Fragment Viability Verified with PI

Quantification of metabolic changes within biological samples is only possible in portions of tissue that are initially viable. Hence, the utility of MP-FLIM was first tested for baseline screening of LTF viability prior to treatment. For this, freshly fragmented CT26 (murine colorectal carcinoma) LTFs were imaged for 2 h following the addition of PI. Using the NAD(P)H autofluorescence intensity [illustrated in grayscale in Fig. 1(a)], single-cell Voronoi segmentation was undertaken as described in Sec. 2. Utilizing the measured fluorescence lifetime data for NAD(P)H and FAD, we calculated an LMR, a metric which can be monitored across time, in which the dynamics have been transformed into our NMS (see Sec. 2) [Fig. 1(b)]. Average values of the NMS, as well as total summed PI intensity, were obtained per each segmented cell region. Using this PI intensity as our ground truth for alive or dead (PI low or high, respectively), the area under the receiver operator characteristic (AUROC) analysis was undertaken using our NMS metric [Fig. 1(c)]. With 10-fold cross-validation, our AUC separation score between live and dead using NMS resulted in a value exceeding 0.9. In addition, this allowed for the calculation of an optimal NMS separation threshold value [$T=0.33\pm 2.5\times 10^{-2}$, see Fig. 1(c)], that is, a single value that correlated best with our binarized PI death indicator for viability screening. Subsequently, using this value, each cell within thirty CT26 fragments was classified as alive (NMS[−]/PI[−]) or dead (NMS[+]/PI[+]), wherein NMS[−] cells have shift values below 0.33 and NMS[+] cells have shift values above 0.33. The total percentage of dead cells quantified via both approaches was compared across each of the individual fragments via linear regression [Fig. 1(d)]—resulting in an $R^{2}=0.974$ (slope = 0.779, intercept = 0.45%, $p\text{ value}<10^{-5}$).

### 3.2 Metabolic Plasticity Is Quantified for Various Cell Death Mechanisms

**Fig 2:**
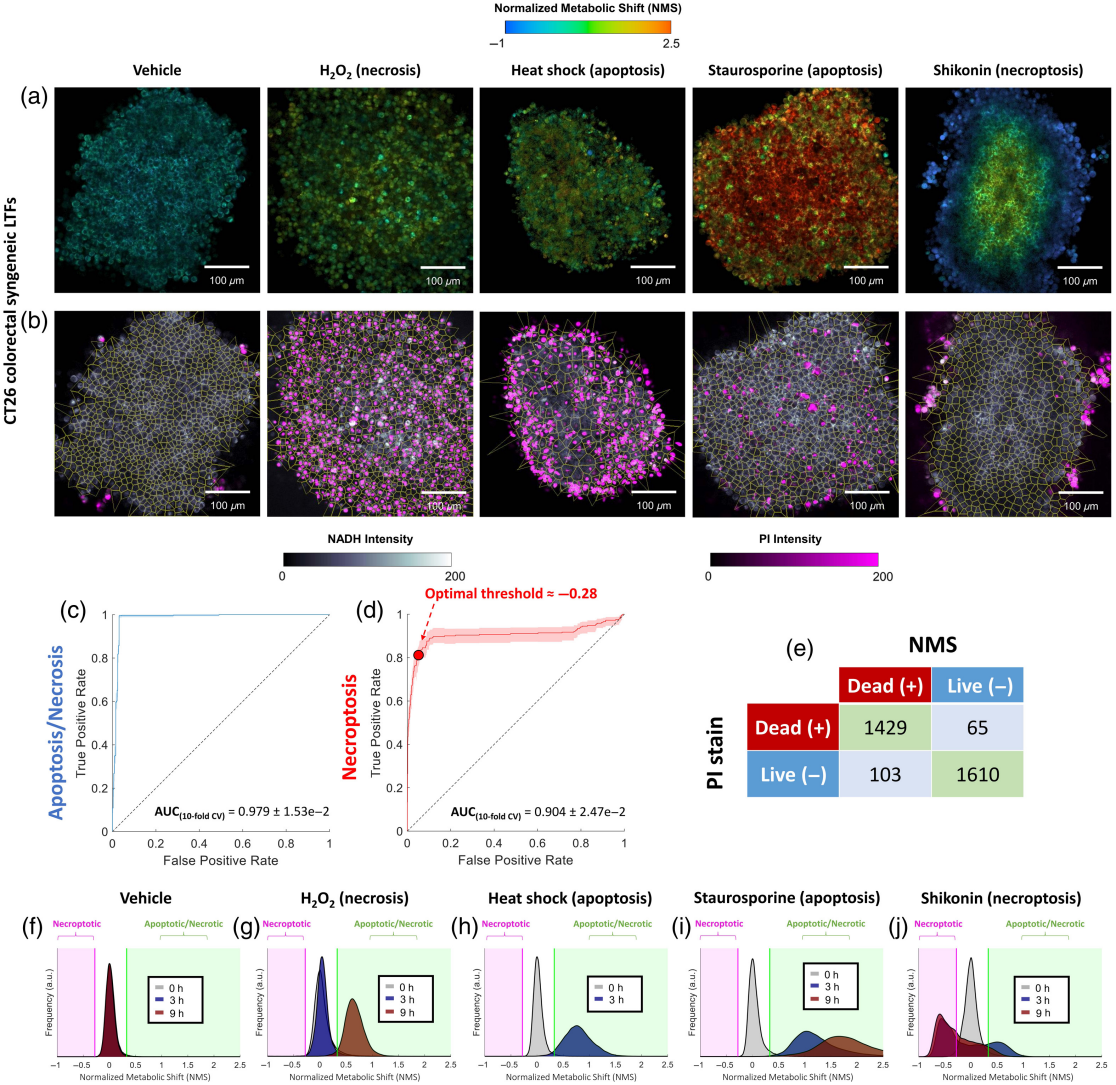
NMS assessment across various mechanisms of cell death in CT26 LTFs—validated against PI (cell death ground truth). (a) NMS images following treatment with different chemicals ($H_{2}O_{2}$, staurosporine, and shikonin) or conditions (heat shock) compared with untreated control (vehicle). (b) Corresponding images of PI exogenous staining. (c) and (d) Ten-fold cross-validation AUROC analysis using averaged NMS per cell region marked alive or dead via PI staining. AUC analysis for all necrosis and apoptosis-inducing agents is shown in panel (c), whereas AUC analysis for shikonin-induced necroptosis is provided in panel (d). (e) Confusion matrix comparison of dead/alive identification using PI stain or by label-free NMS. (f)–(j) KDE distributions of NMS obtained across the entire field of view (FOV) before and after being subjected to treatment. Importantly, photon count thresholding was undertaken to reject pixels with an insufficient signal from subsequent analysis (see Sec. 2).

Cell death can occur along a variety of different pathways, each of which has a different metabolic effect on the cell.^18^^,^^44^ To assess whether shifts in autofluorescence FLIM signatures are measurable across a range of the most commonly observed cell death mechanisms, a multi-treatment assay was conducted using our LTFs. $H_{2}O_{2}$ has been used as a necrosis-inducing agent for validation of FLIM assays in recent literature.^18^^,^^45^ In addition, staurosporine^18^^,^^19^ and heat shock are both commonly utilized ground-truth treatments for inducing mitochondria-mediated apoptosis. Furthermore, necroptosis (i.e., programmed necrosis) has been widely investigated as a possible death mechanism of interest in the event that cancerous cells are resistant to apoptosis.^46^ For this, shikonin has demonstrated promise as a necroptosis-inducing agent,^47^ the effects of which have recently been probed using autofluorescence FLIM of the red channel attributed to lipofuscin.^30^ Hence, CT26 fragments were either left untreated or treated with $H_{2}O_{2}$, heat shock, staurosporine, or shikonin 2 h after staining with either PI or Caspase 3/7 Red, an exogenous label for apoptosis. Spatial maps of the NMS and corresponding PI images comparing treated and untreated fragments are shown in Figs. 2(a) and 2(b).

**Fig 3:**
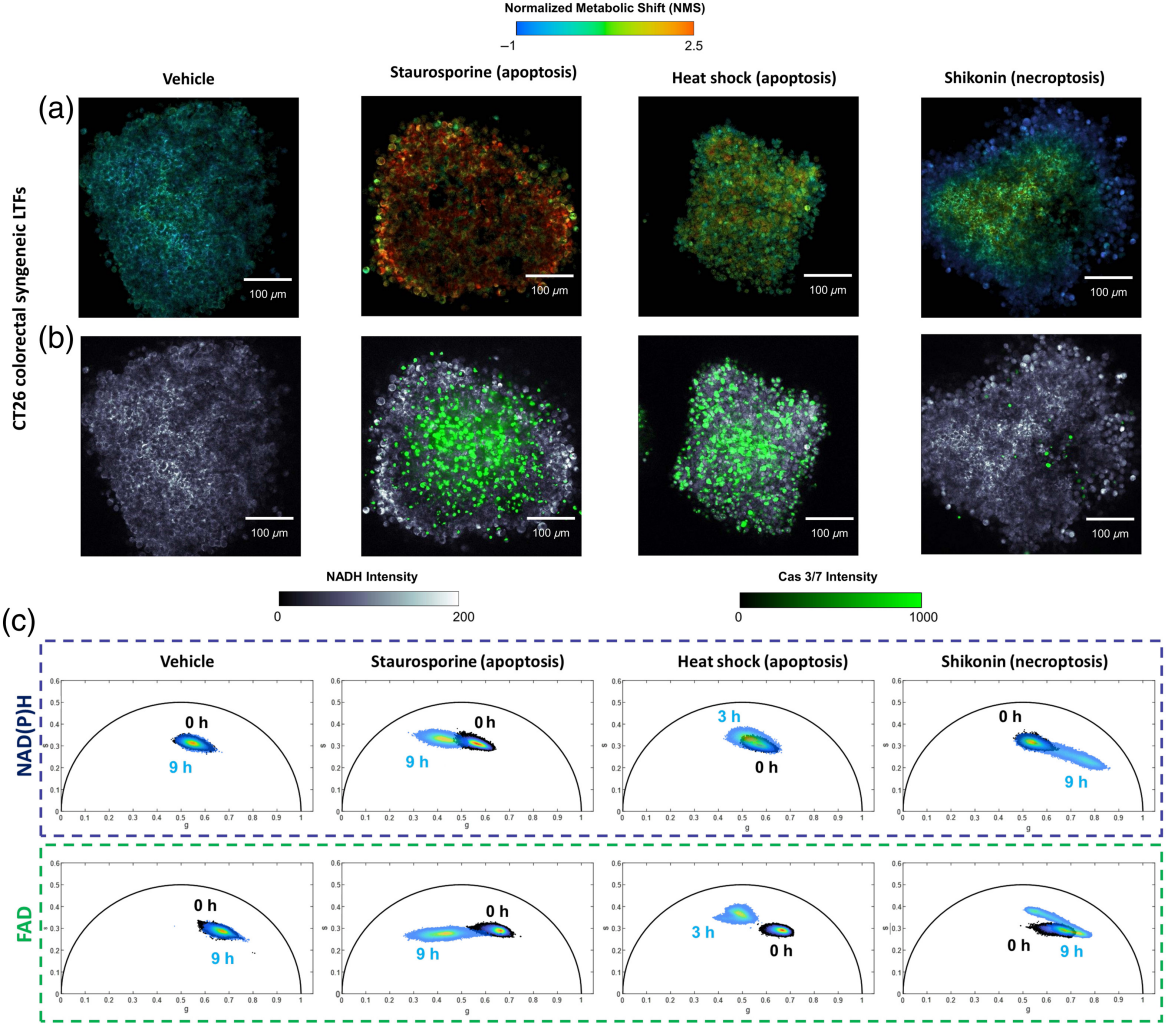
NMS assessment across various mechanisms of cell death in CT26 LTFs—validated against Caspase 3/7 Red (apoptosis). (a) NMS images following treatment with different chemicals (staurosporine and shikonin) or conditions (heat shock) compared with untreated control (vehicle). (b) Corresponding images of Caspase 3/7 Red exogenous staining. (c) NAD(P)H and FAD, and their proportional representation pre- and post-treatment, summarized into phasor plots (described in Sec. 2) where the values in each pixel are plotted, forming a cloud of data points in the plot. Each treatment’s impact on the FLIM signature is marked by a distinct shift in the phasor cloud compared with baseline measurement.

Following treatment with staurosporine, the phasor trajectory (i.e., the line passing through the phasor cloud) was rotated counterclockwise for both NAD(P)H and FAD emission channels, a finding in line with the “long lifetime species” previously observed in HeLa cells after undergoing oxidative stress.^36^ By contrast, heat shock caused both phasor clouds to move along the baseline trajectory toward the longer lifetime reference points, reflecting an increase in the relative concentration of bound NAD(P)H and unbound FAD [Fig. 3(c)]. We found that these changes greatly precede the subsequent death signal observed via positive PI staining, which was observed to stain a greater proportion of cells at later time points (≈24  h), reflecting the predicted poor cell health initially observed via our lifetime imaging. However, most treatments, with the exception of shikonin, killed most observed cells in the fragment in 9 h.

The utility of our microscopy platform’s on-stage incubator (see Sec. 2) for maintaining metabolic homeostasis in the absence of treatment is supported by a lack of observable change in the FLIM signature from 0 to 9 h [Fig. 2(f)]. For quantification of these metabolic signatures, NMS probability distribution functions are provided for each fragment at all three measured timepoints [Figs. 2(f)–2(j)]. Notably, the NMS threshold obtained through AUROC analysis in Fig. 1 holds for $H_{2}O_{2}$-induced necrosis [Fig. 2(g)], as well as staurosporine and heat shock-induced apoptosis [Figs. 2(h) and 2(i)]. However, following treatment with necroptosis-inducing shikonin, the fractional contribution of free NAD(P)H increases significantly (from ≈50% to 75%), causing a large decrease in the measured NMS of cells stained with PI located on the fragment periphery [Figs. 2(a), 2(b), and 2(j)].

The lack of Caspase 3/7 staining confirmed the caspase-independent demise of this necroptotic outermost cell population [Figs. 3(a) and 3(b)]. Single-cell AUROC analysis of untreated and shikonin-treated fragments was undertaken in a similar fashion to that of Fig. 1(c), and the optimal leftmost threshold was determined ($T_{\text{left}}=-0.28$) [Fig. 2(d)]. Altogether, with this threshold and the rightmost one determined through the viability screen (see Fig. 1), three separate ranges in the NMS were established: (i) ${\mathrm{NMS}}_{\text{necroptotic}}=(-\infty ,-0.28)$, (ii) ${\mathrm{NMS}}_{\text{viable}}=[-0.28,0.33]$, and (iii) ${\mathrm{NMS}}_{\text{apoptotic}/\text{necrotic}}=(0.33,\infty )$. This observation clearly highlights the utility of NAD(P)H and FAD FLIM imaging as a live tissue assay for sensitive assessment of drug kinetics.

### 3.3 Assay Is Validated Across Additional Tumor Subtype

**Fig 4:**
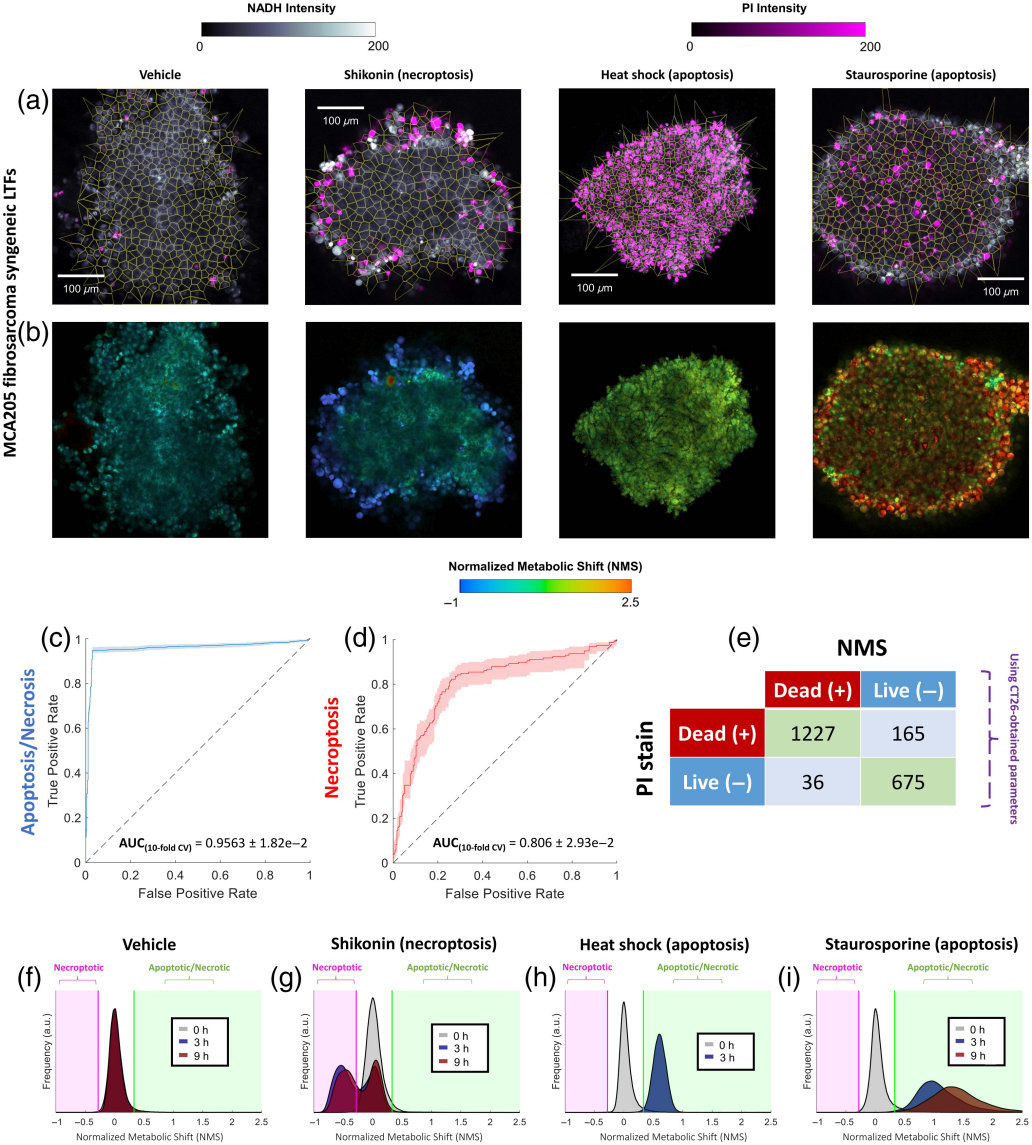
NMS assessment across various mechanisms of cell death in MCA205 LTFs—validated against PI. (a) NMS images following treatment with different chemicals (staurosporine and shikonin) or conditions (heat shock) compared with untreated control (vehicle). (b) Corresponding images of PI exogenous staining. (c) and (d) Ten-fold cross-validation AUROC analysis using averaged NMS per cell region marked alive or dead via PI staining. AUC analysis for all necrosis and apoptosis-inducing agents is shown in panel (c), where AUC analysis for shikonin-induced necroptosis is provided in panel (d). (e) Confusion matrix comparison of dead/alive identification using PI stain or by label-free NMS using values obtained in separate syngeneic tumor models (i.e., CT26—see Figs. 1 and 3). (f)–(i) KDE distributions of NMS obtained across the entire FOV of fragments before and after being subjected to treatment.

FLIM signatures differ across cancer cell types.^42^ To validate whether the NMS shows metabolic perturbations across different cancer models, we extended our multi-treatment assay into the MCA205 murine fibrosarcoma syngeneic line. For this, freshly fragmented MCA205 LTFs were stained with PI or Caspase 3/7 Red dye, and baseline imaging was acquired following 2 h of incubation. Subsequently, these fragments underwent heat-shock treatment, as well as staurosporine and shikonin exposure, as was described previously for CT26 fragments, and were imaged at 3 and 9 h post-treatment. Resulting PI and NMS maps, as well as single-cell Voronoi segmentation overlays, are shown in Figs. 4(a) and 4(b). The corresponding NMS probability distribution functions are provided [Figs. 4(f)–4(i)], where the left and right thresholds (pink [necroptotic] and green [apoptotic/necrotic], respectively) determined with the previous CT26 LTF experiments were used for binary classification of live or dead status per MCA205 cells. Resulting values of accuracy, sensitivity, and specificity (90.4%, 97.5%, and 80.7%, respectively) reflect the high discriminative potential of endogenous FLIM for assessment of cell status. In addition, single-cell AUROC analysis of NMS classification using PI staining as live/dead ground truth was undertaken in a similar fashion as in Fig. 3. The resulting AUC values (${\mathrm{AUC}}_{\text{apoptosis}/\text{necrosis}}=0.956\pm 1.82\times 10^{-2}$ and ${\mathrm{AUC}}_{\text{necroptosis}}=0.806\pm 2.93\times 10^{-2}$) further demonstrate the robustness of this NMS assay across cancer subtypes [Figs. 4(c)–4(e)]. The corresponding phasor density plots and Caspase 3/7 Red validation of caspase-dependent (apoptosis) or independent (necrosis, necroptosis) cell death are provided in Figs. S1–S2 in the Supplementary Material.

### 3.4 Toxic Effects of Chemotherapeutic Efficacy Quantified in LTFs

**Fig 5:**
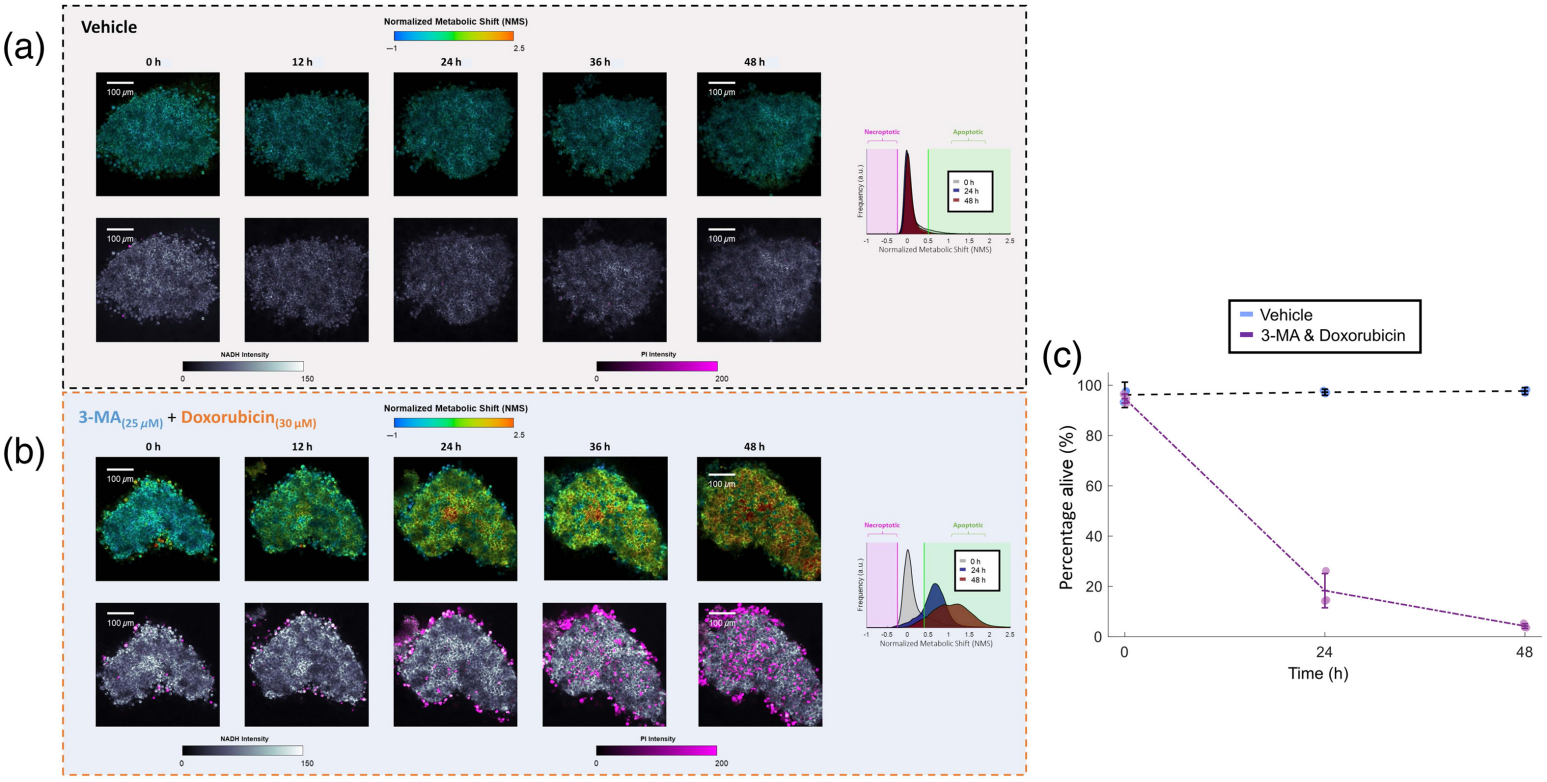
NMS assessment of chemotherapeutic efficacy in CT26 murine LTFs. (a) and (b) NMS and PI images of LTFs treated with either vehicle (a) or with a combination of 3-MA and doxorubicin (b). KDE distributions of NMS obtained across the entire FOV of fragments at baseline and 24 and 48 h are included for both cases. (c) Viability plot across time post-treatment determined via NMS imaging for vehicle and 3-MA/doxorubicin-treated LTFs (n=3 fragments per condition).

Cytotoxic chemotherapy is widely used as a clinical cancer treatment. In contrast to the harsh chemicals and treatments used above, most chemotherapies aim to block cell cycle progression and cause eventual caspase-dependent death in a slower manner. Consequently, the permeability of the nuclear envelope and subsequent PI staining should not be observable until much later timepoints following treatment with traditional chemotherapies compared with the 9 h duration used for the experiments detailed above. Hence, to validate the clinical potential of our platform’s imaging arm, freshly fragmented CT26 LTFs underwent longitudinal NMS imaging following chemotherapy treatment. For this, LTFs were either left untreated or exposed to the anthracycline doxorubicin as well as 3-methyladenine, an autophagy inhibitor that has been shown to enhance the cytotoxic effects of doxorubicin.^48^^–^^50^ Figures 5(a) and 5(b) show the NMS maps across 48 h in 12-h increments for a representative fragment within both groups. High viability is observed, via negligible PI staining as well as a lack of shift in the NMS probability distribution in the untreated fragments over the entire experiment duration, further supporting the effectiveness of our scope incubator and microscopy sample culture conditions for these LTF samples. By contrast, as expected, levels of PI staining eventually increased across time for the chemotherapy treatment group. Importantly, notable increases in PI were left undetected until ∼24  h post-treatment, whereas metabolic shifts in NMS compared with untreated control were observed much earlier [Fig. 5(b), ≈12  h p.t]. In addition, doxorubicin-induced death is attributed mostly to apoptosis, which agrees with the rightward NMS shift observed herein. Using the previously determined NMS thresholds, a percentage of live/dead quantification was undertaken at baseline, 24- and 48-h post-treatment [Fig. 5(c)].

## 4 Discussion and Conclusion

Given the large pool of existing drug options available, the identification of optimal therapy regimens following diagnosis poses an enormous challenge. At present, drugs are oftentimes chosen based on gene mutation status and protein expression obtained via biopsy, and corresponding assessments of drug effectiveness can only take place following weeks of treatment by measurements of tumor burden (i.e., tumor volume). Hence, personalized medical technology capable of accurately identifying therapies for improved response prediction on a case-by-case basis, prior to any initial treatment taking place, is highly sought after.

Toward this aim, we developed and fully characterized a label-free imaging assay based on FLIM and demonstrated its agreement with the conventionally used PI ground-truth death label in live tissue. Specifically, the quantitative information provided through lifetime metabolic imaging of NAD(P)H and FAD autofluorescence enabled sensitive prediction of live/dead status on a single-cell basis using our NMS. With this, highly sensitive LTF baseline viability screening on par with PI staining was demonstrated (AUC>0.9). The robustness of this NMS imaging method was then tested across a variety of cell death mechanisms—such as necrosis, apoptosis, and necroptosis—as well as across two subtypes of syngeneic cancer models. In both cases, the NMS showed high concordance with ground-truth exogenous staining. Moreover, the unique metabolic signature of necroptosis was detected via FLIM for the first time. Surprisingly, this signature was completely different from that of necrosis and apoptosis, which allowed for the determination of three live/dead ranges for our NMS. The optimal threshold values determined through experiments using CT26 murine colorectal LTFs translated when using an entirely different syngeneic cancer model (MCA205 fibrosarcoma) across multiple cell death pathways—confirmed with PI. This evidence supports the notion that, when investigating metabolism in already metabolically transformed tumor tissue, what is important is not the absolute values of the starting condition but the relative metabolic change that occurs following treatment indicating a path toward cell death.

Notably, the unique potential of this approach as an assay to support preclinical drug discovery was clearly highlighted by the observation of differing drug kinetics across treatments. Indeed, staurosporine rapidly caused the highest shift in the normalized LMR—in complete alignment with previously published findings.^19^ $H_{2}O_{2}$ had a similarly devastating effect on the CT26 fragments, which was only observed after the 3-h timepoint. Interestingly, shikonin-induced necroptosis was only observed on the outermost periphery of fragments treated with this therapy, even at 9 h post-treatment. This difference, which is also observed in the MCA205 cancer model, suggests that shikonin’s potential to penetrate into highly dense solid tumor tissue may be limiting. Further investigation of this finding is ongoing.

In addition, to more directly demonstrate clinical potential, the NMS imaging assay was used to assess LTF viability for more than a duration of 48 h in the absence of treatment or following exposure to the chemotherapy doxorubicin. Untreated fragments were measured as having high viability (>90%) across the entire experiment duration, validating our apparatus’ on-scope LTF culture conditions. By contrast, LTFs treated with chemotherapy gradually developed a rightward shift in measured NMS profiles, indicative of apoptosis, an expected finding using such therapies in combination with autophagy inhibition.^50^ A similar viability trend was observed using PI, but only at much later timepoints (≈24  h) compared with the normalized metabolic shift (≈12  h). Given that metabolic changes in response to treatment are expected to greatly precede the breakdown of cell membranes and subsequent PI-positive staining, this finding is not at all surprising but nevertheless reassuring.

It should be noted that, with the label-free and nondestructive nature of this imaging paradigm, all other end-point assays can be used downstream to NMS assessment. For this, developments to pair our NMS imaging assay with other powerful cellular phenotyping and biomarker assessment methods are ongoing.

Though the approach described herein demonstrates promise, it is not without limitation. For instance, LTF samples herein were retrieved from dense syngeneic murine tumors, which are mostly comprised of cancer cells. This is shown in Fig. S3 in the Supplementary Material, which demonstrates how monocellular LTFs retrieved from separate syngeneic murine models can be normalized via NMS without issue. Hence, one can expect this assay to provide accurate readouts of cytotoxicity in samples such as monocellular spheroid or organoid models of cancer. However, for samples that are expected to possess a much higher diversity in cellular composition (e.g., human tissue), this baseline normalization would have to be undertaken either (i) after identification of cancerous cell populations is realized (assuming only cytotoxicity of tumor cells is of interest to the end-user) or (ii) on all cell types independently. For the latter, NMS analysis would be conducted via normalization to the mode of each cell type’s LMR [see Eq. (4)] independently in a population-wide fashion following multi-class segmentation. Previous work has demonstrated the utility of endogenous FLIM for identifying cancer cell populations based on expected increases in glycolysis due to the Warburg effect.^51^^,^^52^ Hence, future work employing an approach such as NMS for assessing complex multicellular samples will likely benefit heavily from using metabolic FLIM for the identification of cancerous tissue prior to longitudinal monitoring of cytotoxicity.

Moreover, it is important to note that though we limited our preliminary work herein to a single plane within our LTF samples, 2P microscopy is well suited for capturing data in 3D. By imaging multiple z planes, a larger portion of the tissue volume would be assessed, and the statistical rigor would be improved via quantification across a larger total number of cells. We expect that this 3D insight will be particularly valuable in highly heterogeneous LTFs, such as those derived from patient-derived xenograft and primary human tissues. In addition, beyond enabling the measurements of cellular viability and drug-induced cytotoxicity demonstrated herein, future work will incorporate other powerful metabolic indicators of immunotherapy response—such as T cell activation (i.e., switch from oxidative phosphorylation to glycolytic metabolism) following immune checkpoint inhibition.^53^^,^^54^

## Supplementary Material



## Data Availability

Data underlying the results presented in this paper are not publicly available but may be obtained from the authors upon reasonable request.
