# Supplementary material for: Label-free fluorescence lifetime imaging for the assessment of cell viability in living tumor fragments
Source: J Biomed Opt. 2024 Jun 14;29(Suppl 2):S22709. doi: 10.1117/1.JBO.29.S2.S22709 (PMC11177118; doi:10.1117/1.JBO.29.S2.S22709)
Supplement: Supplementary file 1 [file JBO_029_S22709_SD001.pdf]

Appendix A: Supplemental materials

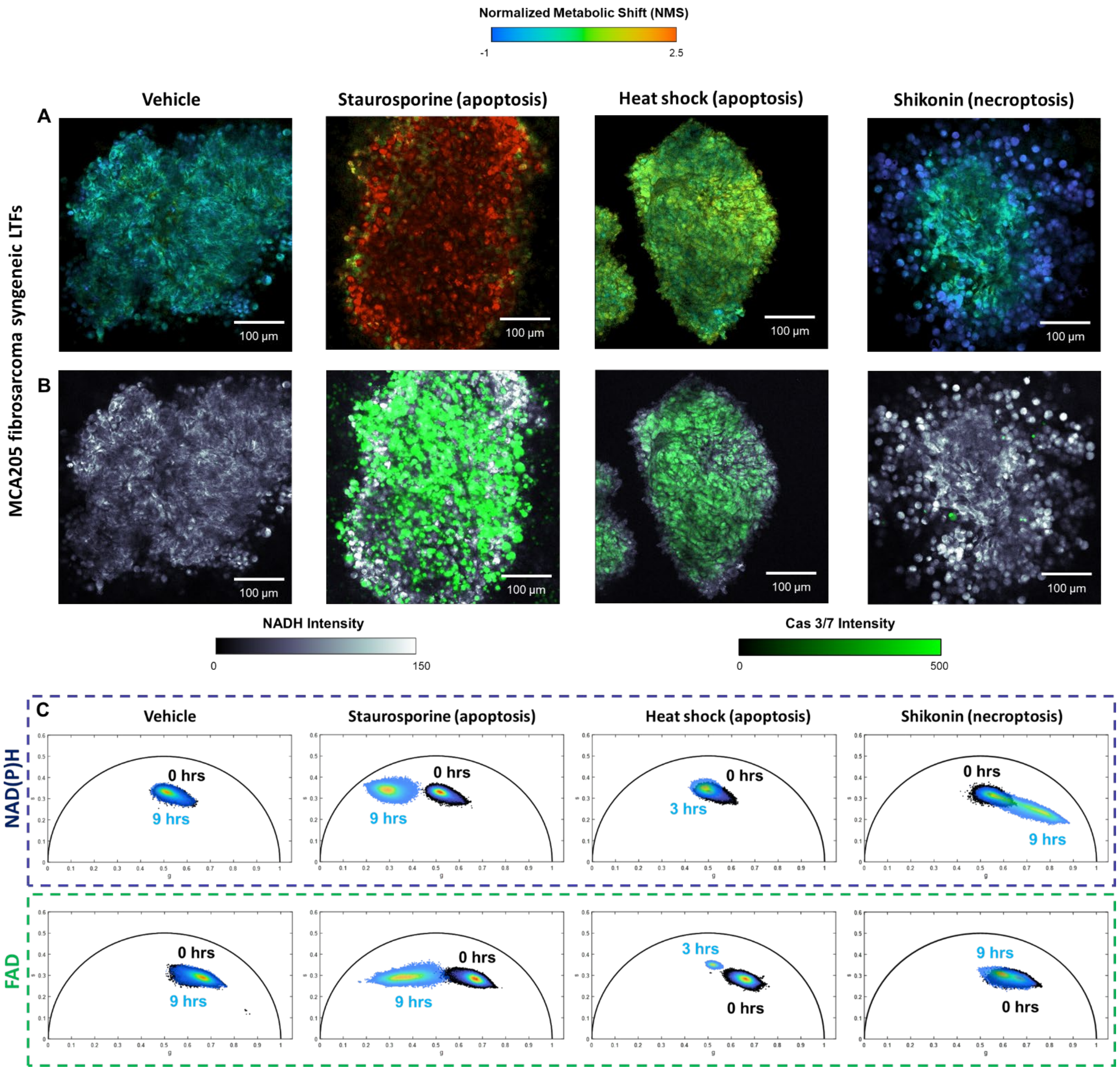

Fig S1. NMS assessment across various mechanisms of cell death in MCA205 LTFs – validated against caspase 3/7 (apoptosis). A: NMS images following treatment with different chemicals (staurosporine and shikonin) or conditions (heat shock) compared to untreated control. B: Corresponding images of caspase 3/7 red exogenous staining. C: Corresponding density plots of NAD(P)H and FAD phasor-FLIM pre and post-treatment.

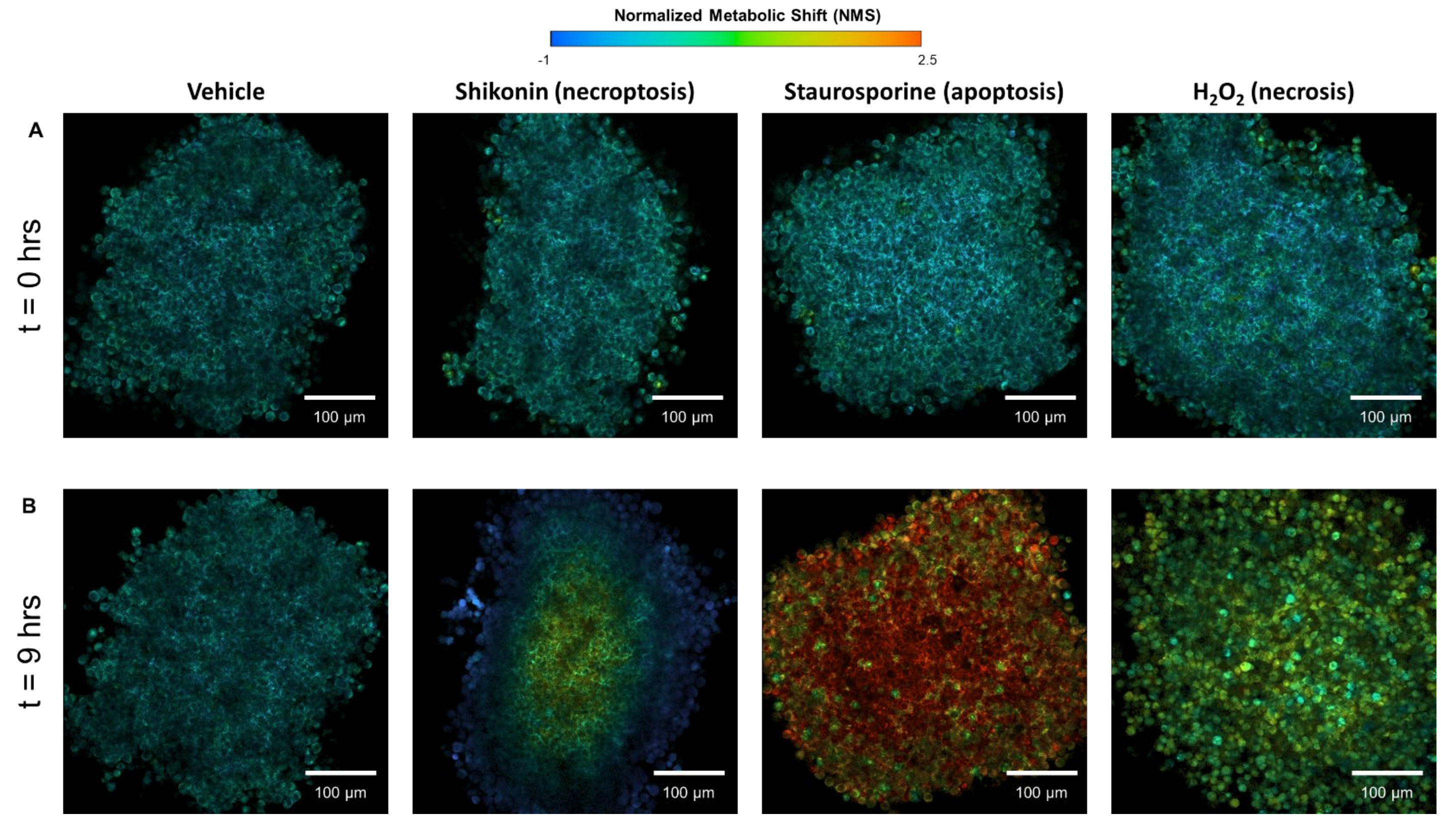

Fig S2. NMS detects shifts in metabolism dependent on baseline normalization. A: NMS maps of CT26 fragments before treatment with death-inducing chemicals from Fig. 2. B: Corresponding NMS maps of the exact same LTF imaged 9 hours later. Treatments are listed above each column.

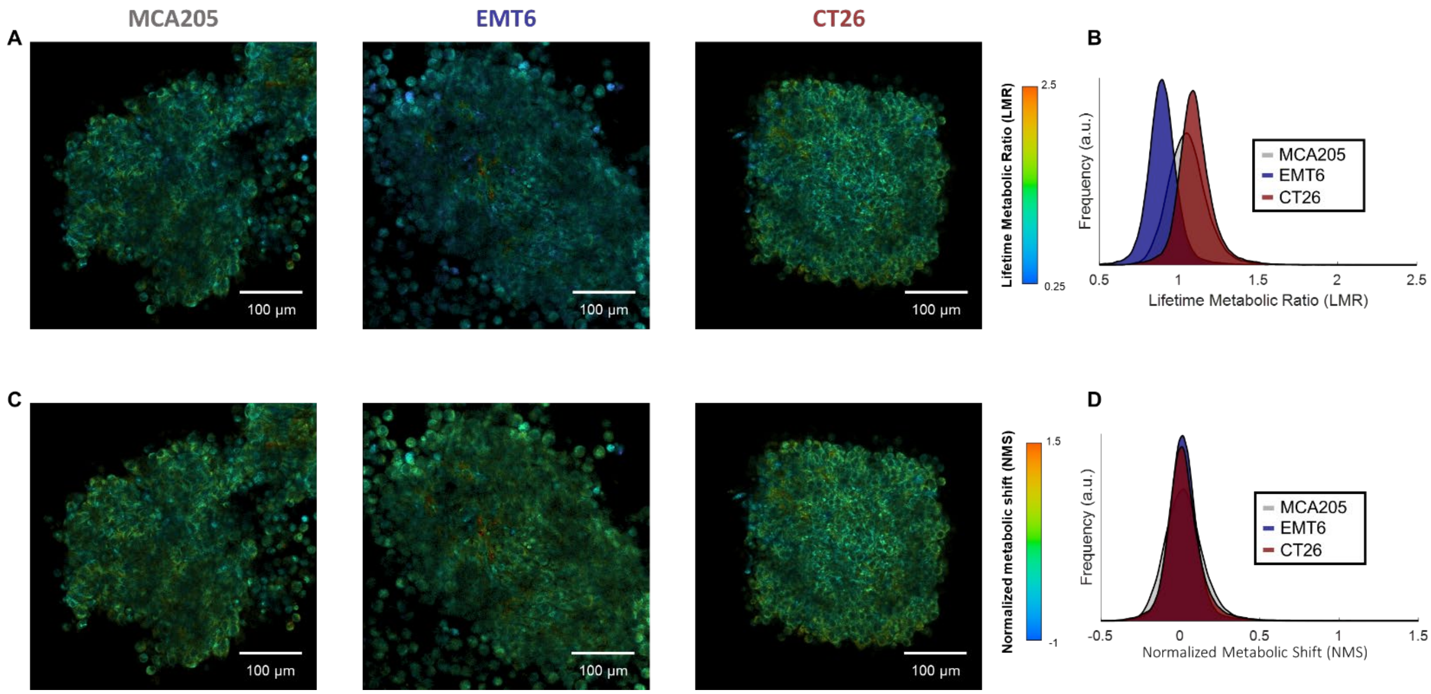

Fig S3. Resting LMR across multiple syngeneic cancer LTFs compared with NMS. A: LMR images of MCA205, EMT6 and CT26 at baseline. B: KDEs of LMR across the entire fragment FOV for all three tissue types. C: Fragments from panel A normalized to their NMS counterparts. D: KDEs of NMS (i.e., normalized versions of panel B) across the entire fragment FOV for all three tissue types. Each NMS distribution is shifted to center around the value zero.
